# Formulæ for the Internal Administration of Chloroform

**Published:** 1856-04

**Authors:** M. Dannecy


*Formulæ for the Internal Administration of Chloroform.* By M. Dannecy.

(L'Union Med., April, 1855.)

M. Dannecy employs oil to dissolve the chloroform. The formula he uses is as follows: Take of pure chloroform, 2 grammes; oil of sweet almonds, 8 grammes; gum arabic, 4 grammes; syrup of orange flowers, 30 grammes; distilled water, 60 grammes. Mix the oil with the chloroform, and make with the mixture an oily draught in the ordinary way. When gum alone is employed to suspend chloroform in a draught, separation of the latter sooner or later takes place; and where alcohol is used, as by many practitioners, in proportion of one part chloroform to four parts alcohol, an excitant is introduced which may not be desirable, and if the quantity of chloroform prescribed be considerable, this objection is a serious one. The advantages which M. Dannecy sees in his formula are: 1. That a perfectly homogeneous and stable mixture is produced, whatever be the proportion of chloroform prescribed; 2. That no excitant like alcohol is introduced into potions which are most frequently intended to be calmative; 3. That it dispenses with every kind of precaution on the part of the patient, or those who have the care of him in administering the remedy. He thinks, further, that the mixture of the chloroform with the oil, without any detraction from the limpidity of the latter, is a test of the purity of the chloroform.

The Commission of the Société de Pharmacie, while admitting M. Dannecy's formula as rational, proposes the following: Chloroform, 2 or 4 grammes; sugar, 12 grammes; gumarabic, 5 or 10 grammes; water, 100 grammes. Th chloroform is added to the sugar in a mortar, then the gum is added, and lastly, by degrees, the water. M. Deschamps, in commenting on the several formulæ which have been suggested, considers that of the Commission as preferable both to that of M. Dannecy and of M. Wahu, who dissolved chloroform in three or four parts of alcohol, and then mixed it with a solution of gum, on the ground that submitting all patients to the action of much alcohol or oil is not a matter of indifference. It is true that, after a time, a whitish floculent deposit takes place, but a little shaking restores the appearance of the mixture. M. Deschamps proposes another formula, viz: Chloroform, 2, 4, 6, &c., grammes; syrup, 30 grammes; yolk of one egg; water, 150 grammes. Dilute the yolk of egg with the water, and strain; weigh the syrup, then the chloroform; add the strained liquor, and shake the whole together.—*Med. Chir. Review.*

